# Antiosteoporotic effects of Polycan in combination with calcium lactate-gluconate in ovariectomized rats

**DOI:** 10.3892/etm.2014.1793

**Published:** 2014-06-20

**Authors:** JAE-SUK CHOI, JOO WAN KIM, KI YOUNG KIM, HYUNG-RAE CHO, IN SOON CHOI, SAE KWANG KU

**Affiliations:** 1 RIS Center, Industry-Academic Cooperation Foundation, Silla University, Busan 702-701, Republic of Korea; 2 Glucan Corporation Research Institute, Marine Bio-Industry Development Center, Busan 619-912, Republic of Korea; 3 Department of Biological Science, Silla University, Busan 702-701, Republic of Korea; 4 Department of Anatomy and Histology, College of Oriental Medicine, Daegu Haany University, Gyeongsan 712-715, Republic of Korea

**Keywords:** calcium lactate-gluconate, osteoporosis, Polycan, rat

## Abstract

The aim of the present study was to investigate the optimum composition of Polycan (β-glucan complex) and calcium lactate-gluconate (CaLG) that exhibited the most beneficial effects in ovariectomy (OVX)-induced osteoporotic rats. Polycan and CaLG single formulas (100 mg/kg each), and three doses (50, 100 and 200 mg/kg) of three mixed formulas [polycan:CaLG (PCLG)=1:99, 5:95 and 10:90] were orally administered once a day for 84 days. The effects of the test materials were compared with those of a risedronate sodium-treated group. OVX resulted in an increase in body weight, decreased bone formation, elevated serum osteocalcin levels and urine deoxypyridinoline/creatinine ratio, as well as decreased serum bone-specific alkaline phosphatase levels, femur indices, bone mineral content, bone mineral density and failure load. However, these OVX-induced osteoporotic changes markedly decreased following the administration of the test materials. Continuous oral treatment of Polycan or CaLG single formulas and the PCLG mixed formulas preserved bone mass and strength. The PCLG 10:90 mixed formula exhibited the most favorable synergistic antiosteoporotic effects in the OVX-induced osteoporotic rats as compared with equal doses of the Polycan or CaLG single formulas.

## Introduction

Osteoporosis is a metabolic bone disease resulting from a disturbance of normal bone remodeling that shifts the balance to bone resorption over bone formation, causing bone loss and fractures (1). Osteoporosis affects ~25 million individuals in the United States alone and it is estimated that females >50 years-old possess an 11–18% lifetime risk of suffering a hip fracture (2). Numerous attempts to develop novel agents capable of preventing and/or treating bone diseases have been investigated (3). Currently, antiresorptive agents are extensively used, however, more highly efficacious resorptive inhibitors with excellent safety and efficacy are required. Anabolic agents that stimulate bone formation are less well investigated in comparison to antiresorptive agents (4). With advances in the understanding of osteoblast differentiation and bone formation, continuous trials to develop anabolic agents have been performed (5,6).

The estrogen-deficient ovariectomy (OVX) model is useful for the evaluation of osteoporotic drugs, as several parameters are decreased by OVX within 4–6 weeks of surgery. The OVX rat model was selected for the present study as it shares numerous similarities with postmenopausal bone loss (7) and is recommended by the US Food and Drug Administration as a test species for evaluating the long-term skeletal safety and efficacy of osteoporosis therapies (8).

Calcium (Ca) salts have a reported therapeutic benefit against osteoporosis (9). Ca supplements are an important alternative source of Ca and minimize bone loss during aging (10,11). The bioavailability of Ca varies in Ca supplements and can be affected by disintegration, solubility, chelate formation and food-drug interactions (12,13). Polycan, a commercial β-glucan obtained from *Aureobasidium pullulans* SM-2001, is primarily comprised of β-1,3/1,6-glucan and other organic materials, including amino acids, mono- or di-unsaturated fatty acids (linoleic and linolenic acids) and fibrous polysaccharides (14). Polycan has been reported to induce antiosteoporotic effects via inhibiting bone loss, accelerating bone formation (15), promoting fracture healing (16) and exhibiting anti-inflammatory effects (17).

Therefore, a mixed formula consisting of water soluble Ca salts and Polycan is expected to exhibit synergic antiosteoporotic effects, providing a potential novel preventive or therapeutic regime for osteoporosis. In the present study, compositions of Polycan and calcium lactate-gluconate (CaLG) were investigated with the aim of identifying a composition that exhibited the most beneficial effects and synergism in OVX-induced osteoporotic rats. Polycan and CaLG single formulas (100 mg/kg each), and three doses (50, 100 and 200 mg/kg) of three mixed formulas [polycan:CaLG (PCLG)=1:99, 5:95 and 10:90] were orally administered daily to OVX osteoporotic rats for 84 days. Changes in body weight (BW), femur indices, Ca and inorganic phosphorus (P) content, bone mineral density (BMD), failure load (FL), histological profiles and histomorphometrical analyses were assessed. The results of the test materials were compared with those of risedronate sodium (5 mg/kg), a pyridinyl bisphosphonate that binds to bone hydroxyapatites and inhibits osteoclast-mediated bone resorption (18).

## Materials and methods

### Animals

A total of 140 Sprague-Dawley specific pathogen-free female rats (six-weeks old; Japan SLC, Inc., Komagane, Japan) were allocated into polycarbonate cages (four per cage) in a temperature (20–25°C) and humidity (30–35%) controlled room. The rats were allowed to acclimatize for 12 days. The light:dark cycle was 12 h and food (Samyang Foods Co., Ltd., Wonju, Korea) and water were supplied *ad libitum*. OVX-induced osteoporotic rats (n=132) were used for the study, with eight rats used as the sham controls. Eight rats per group (total 14 groups; n=112) were selected based on their BW during the first week following OVX surgery. Briefly, excluding overweight and underweight rats, the rats were grouped into a total of 14 groups. First, after arranging the rats in order of weight, the heaviest 14 rats were randomly assigned to each of 14 groups. The 14 next heaviest rats were then randomly assigned to each of the 14 groups. Therefore, 112 rats were randomly assigned to each of the 14 groups. The animal experiments were performed in accordance with the US National Institutes of Health Guidelines for the Care and Use of Laboratory Animals (19) and the study was approved by the Institute of Laboratory Animal Resources of Daegu Haany University (Gyeongsan, Korea).

### Drug preparation and administration

Polycan and CaLG were supplied by Glucan Corp. (Busan, Korea). Polycan and CaLG single formulas (100 mg/kg each), and three doses (50, 100 and 200 mg/kg) of three mixed formulas [polycan:CaLG (PCLG)=1:99, 5:95 and 10:90] were dissolved in distilled water and orally administered at a volume of 5 ml/kg, daily for 84 days from the first week following OVX. Risedronate sodium (Pharmaceutical Works Polpharma S.A., Starogard, Poland) was dissolved in distilled water and orally administered by gastric gavage at a concentration of 5 mg/kg.

### OVX surgery

Bilateral OVX was performed under Zoletil (Virbac, Carros, France) anesthesia in all the OVX groups, as previously described (11). In the sham controls, the bilateral ovaries were exposed, but not removed, and the incision was closed with skin sutures.

### BW changes

Changes in BW were calculated one day prior to OVX, during OVX, six days following OVX, at the initiation of administration and at each subsequent week using an automatic electronic balance (Precisa Gravimetrics AG, Dietikon, Switzerland). During OVX and at the initiation of administration and termination of treatment, the experimental animals were fasted overnight (water was not included; ~12 h) to minimize BW changes due to feeding.

### Assessment of bone weight

Animals were sacrificed by exsanguination under anesthesia and the wet and dry weights of the right femur were calculated, as previously described (15). Following weighing, the bones were measured using an electronic digital caliper (Mitutoyo Corp., Kawasaki-shi, Japan).

### Blood and urine collection

For blood analysis, 10-ml blood samples were collected from the vena cava at sacrifice and the serum was separated. All serum samples were frozen at −75°C prior to use. For urinalysis, urine was collected over 24 h following the final treatment dose and centrifuged (Thermo Scientific Sorvall Legend Mach 1.6R; Thermo Fisher Scientific Inc., Waltham, MA, USA) at ~600 × g for 10 min to remove any sediments.

### Serum osteocalcin and bone-specific alkaline phosphatase (bALP) level measurement

Serum osteocalcin levels were detected by radioimmunoassay using an Osteocalcina Myria kit (Technogenetics, Milan, Italy) and a gamma counter (COBRA II, Packard, USA). Serum bALP levels were detected via enzyme immunoassay (EIA) using a Metra™ bALP kit (Quidel Corp., San Diego, CA, USA) and an enzyme-linked immunosorbent assay (ELISA) reader (Sirio S; Radim SpA, Pomezia RM, Italy).

### Urine deoxypyridinoline (Dpd)/creatinine ratio measurement

Urine Dpd was detected using an EIA with a Metra™ DPD kit (Quidel Corp.) and an ELISA Reader (Sirio S; Radim). The urine creatinine levels were detected via a Jaffe method using Sicdia creatinine reagents (Shin Yang Chemical Co., Ltd., Korea) and an automated urine analyzer (Toshiba 2000FR; Toshiba, Tokyo, Japan). The Dpd/creatinine ratio was calculated as follows: Dpd/creatinine ratio (nM/g/day) = (Dpd levels/creatinine levels).

### Assessment of bone mineral content (BMC), BMD and FL

BMC was measured in the tibias. Briefly, the tibias were dried at 120°C for 8 h, carbonized at 800°C for 6 h in a furnace (6000, Barnstead/Thermolyne, Dubuque, IA, USA) and dissolved in nitric acid. In the diluted solution, the Ca and P contents were calculated using orthocresolphthalein complexon (Sigma-Aldrich, St. Louis, MO, USA) and enzyme methods (mg/g bone). The BMD was measured in the femur using dual-energy X-ray absorptiometry (g/cm^2^) bone strength was detected as failure load (FL). FL of the midshaft region of the right femur was measured in newtons by a three-point bending test to failure using a computerized testing machine (Instron 6022; Instron, Canton, MA, USA; speed 20 mm/min) (15).

### Histological procedures

The left femur was separated, fixed in 10% neutral buffered formalin and decalcified for five days. The samples were then embedded, sectioned (3–4 μm) and stained with hematoxylin and eosin. Histomorphometry analysis was performed for bone mass, structure and resorption in a uniform area of the epiphyseal regions (growth plate regions were excluded) using an automated image analyzer (DMI-300; DMI, Korea). Cortical bone thickness was measured in the epiphyseal neck and mid-shaft regions. For bone mass and structure analysis, the trabecular bone volume (TBV), thickness (Tbt), number (Tbn) and length (Tbl), as well as the cortical bone thickness (Cbt), were measured as previously described (2). Generally, the degree of osteoporosis is measured by the osteoclast number (Ocn) on the bone surface (cortical bone), or the number of eroded surfaces. The problem caused by osteoporosis is fractures. Therefore, in this study, determining Ocn in the epiphyseal region (metaphyseal area) where fractures sometimes occur, as well as in the midshaft of trabecula bone where numerous fractures occur, we also evaluated the suppressive effects of polycalcium on bone destruction. For the assessment of bone resorption, the Ocn in uniform regions of the epiphyseal (number/epiphyseal) and the osteoclast cell surface/bone surface (OS/BS) were detected as previously described (20).

### Statistical analysis

The results are expressed as the mean ± SD. Multiple comparison tests for the different dose groups were conducted. Variance homogeneity was examined using the Levene test. If the Levene test indicated no significant deviations from the variance homogeneity, the obtained data were analyzed by one-way analysis of variance followed by the least-significant difference multi-comparison test to determine which pairs of group comparison were significantly different. In the cases where significant deviations from the variance homogeneity were observed with the Levene test, a non-parametric comparison test, Kruskal-Wallis H test, was conducted. When a significant difference was observed with the Kruskal-Wallis H test, the Mann-Whitney U-Wilcoxon Rank Sum W test was conducted to determine the specific pairs of group comparison which were significantly different. Statistical analyses were conducted using SPSS for Windows (Release 14K; SPSS, Inc., Chicago, IL, USA). P<0.05 was considered to indicate a statistically significant result.

## Results

### BW assessment

Significant increases (P<0.01) in BW were detected in all the OVX groups between day 7 and 14 following initial administration, as compared with the sham control group. In addition, the BW of all the ovariectomized rats significantly increased during the administration of test material when compared with the sham control rats. However, no significant differences in BW were detected between the formula-administered groups and the OVX control group (data not shown).

### Bone weight assessment

Although a significant decrease (P<0.01) in the relative wet femur weight was detected in the OVX group when compared with the sham control group, significant increases (P<0.01 or P<0.05) in the relative wet bone weights were restricted in the risedronate sodium- and PCLG 1:99 200 mg/kg-treated rats as compared with the OVX control rats. A significant decrease (P<0.01) in the absolute and relative ash femur weights was detected in the OVX control group as compared with the sham control group. The ash femur weight increased in the formula-administered rats when compared with the OVX control rats. Among the three types of mixed formulas, only PCLG 10:90 exhibited favorable synergistic effects against OVX-induced bone weight loss when compared with equal doses of the Polycan single formula. More potent inhibitory effects on bone weight loss were detected in the PCLG 10:90 mixed formula 50 mg/kg-treated rats as compared with the Polycan single formula 100 mg/kg-treated rats (Table I).

### Serum biochemistry

Serum osteocalcin levels in the OVX control group significantly increased (P<0.01), while the serum bALP levels significantly decreased (P<0.01) when compared with the sham control group. However, a significant decrease (P<0.01 or P<0.05) in serum osteocalcin levels with an elevation in bALP levels was observed in all the treatment groups with the exception of the risedronate sodium-treated group, in which a significant decrease (P<0.01) in serum osteocalcin levels was detected with no changes to serum bALP levels. Among the three types of mixed formulas, only the PCLG 10:90 formula exhibited favorable synergistic effects against OVX-induced serum osteocalcin and bALP level changes when compared with equal doses of the CaLG single formula. Similar inhibitory effects on the serum osteocalcin and bALP level changes were detected in the PCLG 10:90 mixed formula 50 mg/kg-treated rats as compared with the CaLG single formula 100 mg/kg-treated rats (Table II).

### Urinalysis assessment

A significant increase (P<0.01) in urine Dpd levels and the urine Dpd/creatinine ratio were observed in the OVX control group when compared with the sham control group. However, these values significantly decreased (P<0.01) in the test material-treated rats when compared with the OVX control. No marked changes in urine creatinine levels were detected in any formula-tested groups compared with the Sham and OVX control groups. Among the three types of mixed formulas, only the PCLG 10:90 mixed formula exhibited favorable synergistic effects when compared with equal doses of the single Polycan formula. Similar inhibitory effects on the urine Dpd levels and a reduction in the Dpd/creatinine ratio were detected in the PCLG 10:90 mixed formula 50 mg/kg-treated rats as compared with the Polycan single formula 100 mg/kg-treated rats (Table III).

### Effects on BMC, BMD and FL

Femur Ca and P contents significantly decreased (P<0.01) in the OVX control group when compared with the sham control group. However, a significant increase (P<0.01) in the femur Ca and P contents was observed in the formula-treated groups when compared with the OVX control group, but with no change to the bone Ca/P ratio. The PCLG 10:90 mixed formula exhibited the most favorable synergistic effects against the OVX-induced decrease in femur Ca and P content when compared with equal doses of the CaLG single formula. Similar inhibitory effects were detected in the PCLG 10:90 mixed formula 50 mg/kg-treated rats as compared with the CaLG single formula 100 mg/kg-treated rats (Table IV).

The BMD of the OVX control group decreased at all the detection points when compared with the sham control group. However, marked increases in the BMD of the measured regions were evident in the formula administration groups when compared with the OVX control group. PCLG 10:90 exhibited a favorable effect against the OVX-induced femur BMD decrease when compared with equal doses of the CaLG single formula. Similar inhibitory effects on the femur BMD decrease were detected in the PCLG 10:90 mixed formula 50 mg/kg-treated rats as compared with the CaLG single formula 100 mg/kg-treated rats (Table V).

The strength (FL) of the femur in the OVX control group decreased compared with the sham control group. However, increases in FL were detected in all the administration groups when compared with the OVX control group. However, only the PCLG 10:90 mixed formula exhibited favorable synergism against OVX-induced femur FL decrease when compared with equal doses of the Polycan single formula. Similar inhibitory effects were detected in the PCLG 10:90 mixed formula 50 mg/kg-treated rats as compared with the Polycan single formula 100 mg/kg-treated rats (Table V).

### Histopathological profile changes

**Figure 1:**
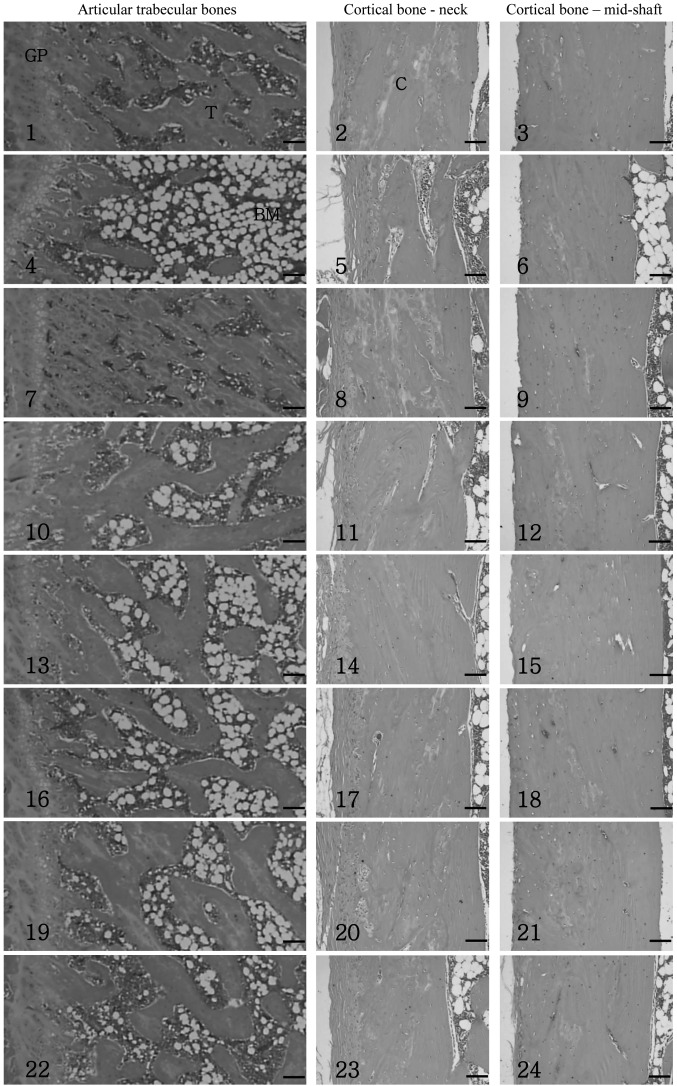

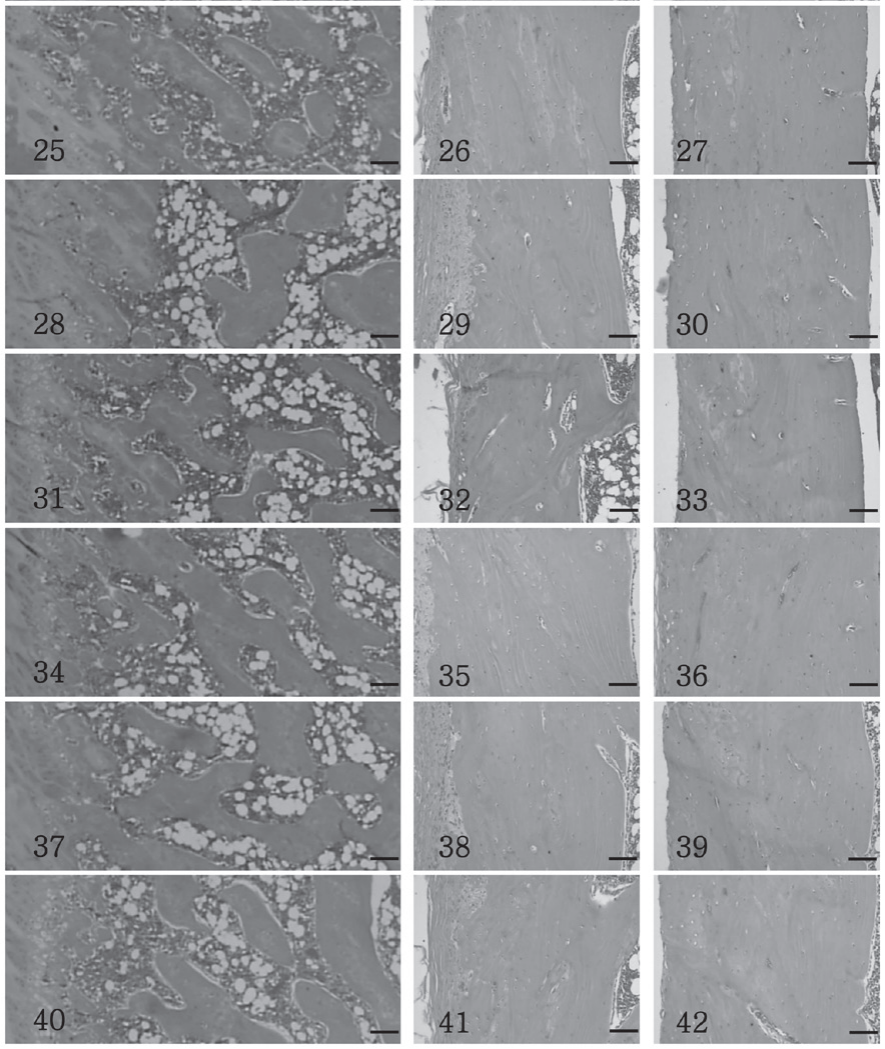
Histological profiles of the femur in osteoporotic rats in the sham control (1–3), OVX control (4–6), risedronate sodium (7–9), Polycan (10–12) and CaLG (13–15) single formula and PCLG 1:99 200 (16–18), 100 (19–21) and 50 mg/kg (22–24) mixed formula groups (hematoxylin and eosin; scale bars, 160 μm). OVX, ovariectomy; CaLG, calcium lactate-gluconate; PCLG, Polycan:CaLG. Histological profiles of the femur in osteoporotic rats in the PCLG 5:90 200 (25–27), 100 (28–30) and 50 mg/kg (31–33) and the PCLG 10:90 200 (34–36), 100 (37–39) and 50 mg/kg (40–42) mixed formula groups (hematoxylin and eosin; scale bars, 160 μm). Relatively well-developed trabecular and cortical bone was observed in the femur of the sham control group. However, a classical osteoporotic histological profile was detected in the OVX control group with a marked histological decrease of trabecular and cortical bone and an increase of connective tissues in the periosteum of the cortical bone, resulting from the resorption of osteoid tissues. These osteoporotic changes were markedly inhibited by treatment with all the test materials used. OVX, ovariectomy; CaLG, calcium lactate-gluconate; PCLG, Polycan:CaLG.

Relatively well-developed trabecular and cortical bone was observed in the femurs of the sham control group. However, a classical osteoporotic histological profile was detected in the OVX control group, including a marked loss of trabecular and cortical bone and increased levels of connective tissue in the periosteum of the cortical bone, resulting from the resorption of osteoid tissues. These osteoporotic changes were markedly inhibited by treatment with the test materials. Among the three types of mixed formulas, only PCLG 10:90 exhibited synergistic effects against the OVX-induced changes when compared with equal doses of the Polycan single formula. Similar inhibitory effects were detected in the PCLG 10:90 mixed formula 50 mg/kg-treated rats as compared with the Polycan single formula 100 mg/kg-treated rats (Fig. 1).

Significant reductions (P<0.01) in the TBV, Tbn, Tbt, Tbl and Cbt (at the epiphyseal and mid-shaft) were detected in the OVX control group when compared with the sham control group. These histomorphometrical indices for bone mass and structure significantly increased in the test material-administered OVX rats when compared with the OVX control rats. However, only the PCLG 10:90 mixed formula exhibited favorable synergistic effects against OVX-induced femur histopathology when compared with equal doses of the Polycan or CaLG single formulas. Similar inhibitory effects were detected in the PCLG 10:90 mixed formula 50 mg/kg-treated rats as compared with the Polycan or CaLG single formula 100 mg/kg-treated rats (Table VI).

Significant (P<0.01) increases in Ocn and OS/BS were detected in the OVX group compared with the Sham control group. This means that the OVX model is good for the evaluation of osteoporosis and that osteoporosis was well induced by OVX in this study. However, decreased Ocn and OS/BS values were observed in the material-administered group compared with the OVX control group. Although a similar Ocn was detected in the risedronate-treated and OVX control groups, the OS/BS significantly (P<0.01) decreased. Only the PCLG 10:90 mixed formula exhibited favorable synergistic effects against the OVX-induced femur histopathological bone resorption changes when compared with equal doses of the Polycan single formula. Similar inhibitory effects were detected in the PCLG 10:90 mixed formula 50 mg/kg-treated rats as compared with the Polycan single formula 100 mg/kg-treated rats (Table VI).

## Discussion

Bone remodeling by osteoblasts and osteoclasts is a crucial determinant of increasing bone mass under pathological conditions, including bone disorders (21). As favorable antiosteoporotic effects of Polycan (15) and CaLG (9) have been reported, the present study aimed to select the optimal PCLG composition that exhibited the most favorable efficacy on OVX-induced osteoporotic rats. Polycan and CaLG single formulas (100 mg/kg each), and three doses (50, 100 and 200 mg/kg) of three mixed formulas [polycan:CaLG (PCLG)=1:99, 5:95 and 10:90] were orally administered daily for 84 days to OVX osteoporotic rats, and changes in the BW, serum osteocalcin levels, urine Dpd/creatinine ratio, femur indices, BMC, BMD, FL and histological and histomorphometrical analyses were assessed.

Alterations in BW were detected in all the OVX groups, which is considered as a general sign of estrogen deficiency (22). In the present study, no marked changes in BW were detected in any of the treated groups when compared with the OVX control group. Therefore, Polycan and CaLG single and mixed formulas were hypothesized to not affect OVX-induced BW increase.

Although it is generally accepted that changes in BW are not a critical index for detecting the efficacy of antiosteoporotic agents (23), an increased ash bone weight is considered a valuable marker of antiosteoporotic activity (24). The PCLG 10:90 mixed formula exhibited favorable inhibitory effects on OVX-induced ash bone weight decrease, providing direct evidence that an appropriate mixture of Polycan and CaLG induces synergistic antiosteoporotic effects.

Although variability in the methods for measuring bone turnover and formation exist among previous studies, serum osteocalcin levels are the generally accepted marker of bone turnover, while bALP levels are considered to demonstrate bone formation (25). However, since osteocalcin is a vitamin K-dependent α-carboxyglutamic acid released by osteoblasts, serum osteocalcin levels are also regarded as an indicator of bone formation (26). In the present study, serum osteocalcin levels in the OVX control group markedly increased compared with those in the sham control group, indicating an increased bone turnover. A marked reduction in the serum osteocalcin levels was detected in all the treated rats when compared with the untreated OVX control, indicating an inhibition of bone turnover. In addition, the decrease in serum bALP levels was significantly inhibited by the Polycan and CaLG single or mixed formulas, providing indirect evidence that the formulas facilitate bone formation. The PCLG 10:90 mixed formula exhibited the most favorable inhibitory effects on the OVX-induced serum osteocalcin and bALP level changes when compared with the single Polycan or CaLG formulas, and thus achieved the most promising synergistic antiosteoporotic effects.

Urine Dpd is a marker of bone resorption that is sensitive to creatinine, an indicator of kidney status (27). The Dpd/creatinine ratio was employed in the present study as an index of bone resorption during osteoporosis (25). The urine Dpd/creatinine ratio significantly increased in the OVX rats, but was inhibited by the formula treatments, indicating that the test materials inhibited the bone resorption induced by OVX. Similarly, the PCLG 10:90 mixed formula exhibited the most favorable inhibitory effects as compared with the single formulas of Polycan or CaLG.

Generally, the BMC significantly decreases during osteoporosis. With regard to BMC, the Ca and P contents represent the most markedly decreased mineral contents during osteoporosis, while the Ca/P ratio does not generally change (28). The Polycan and CaLG single or mixed formulas significantly increased the content of Ca and P in the femur, indicating that the formulas preserve bone mass and are likely to increase bone strength. In addition, favorable inhibitory effects on OVX-induced BMC decreases were detected with the PCLG 10:90 mixed formula as compared with equal doses of the Polycan or CaLG single formulas.

BMD is a marker of bone quantity and generally decreases in osteoporotic animals. The BMD of bone provides reliable information regarding the efficacy of antiosteoporotic agents (29), thus, provides a diagnostic profile of bone quantity (30). All the test materials administered in the present study demonstrated favorable inhibitory effects on the decrease in BMD induced by OVX, regardless of the detection regions.

The FL directly indicates cortical bone strength (31) and is an important indicator of the efficacy of antiosteoporotic agents (32). All the test materials administered in the present study exhibited favorable effects on bone strength. In particular, the PCLG 10:90 mixed formula demonstrated the most favorable inhibitory effects on OVX-induced BMD and bone strength decreases.

The efficacy of various antiosteoporotic agents has been evaluated through bone histology analysis (33,34). Microscopic bone analysis was used to assess bone morphology (31,35). In the osteoporotic animals, the histological profiles were clearly altered regardless of cause, particularly in the trabecular and cortical bone. All the test materials exhibited clear inhibitory effects on these histological changes in the OVX-induced osteoporotic rats.

During osteoporosis, a number of histomorphometrical indices for bone mass decrease, while bone resorption generally increases, providing reliable information to predict the efficacy of antiosteoporotic agents (36). Following treatment with the Polycan and CaLG single or mixed formulas, changes in the histomorphometrical indices for bone mass, structure and resorption were markedly inhibited. These observations provide evidence of the favorable antiosteoporotic effects that these formulas exhibit. The PCLG 10:90 mixed formula demonstrated the most favorable inhibitory effects on the OVX-induced histopathological changes as compared with the single formulas of Polycan or CaLG. Calcium is critical for improving bone health, and its intake is generally recommended. However, the consumption of sufficient calcium is not the only factor required for preventing osteoporosis or bone fracture. If patients receive polycalcium, intake of less than the daily recommended amount of calcium results in suppression of bone destruction and prevention of fractures (FL growth).

## Figures and Tables

**Table I:** Femur weights after 84 days of repeated oral administration of test materials in osteoporotic rats.

	Wet weights		Ash weights	
		
Groups	Absolute (g)	Relative (% of BW)	Absolute (g)	Relative (% of BW)
Controls				
Sham	0.839±0.048	0.324±0.016	0.364±0.018	0.141±0.010
OVX	0.861±0.042	0.264±0.019^a^	0.324±0.015^a^	0.099±0.006^a^
Risedronate sodium	0.893±0.086	0.297±0.032^b^^c^	0.390±0.039^b^^c^	0.129±0.011^b^^c^
Single formula (100 mg/kg)				
Polycan	0.889±0.067	0.277±0.023^a^	0.355±0.020^d^	0.111±0.007^a^^d^
CaLG	0.910±0.064^b^	0.282±0.024^a^	0.356±0.023^c^	0.110±0.008^a^^d^
Polycan:CaLG mixed formula (1:99; mg/kg)				
200	0.916±0.056^b^	0.292±0.027^b^^d^	0.362±0.023^c^	0.115±0.004^a^^c^
100	0.886±0.047	0.282±0.033^a^	0.349±0.024^d^	0.110±0.004^a^^c^
50	0.888±0.037	0.280±0.015^a^	0.354±0.021^d^	0.112±0.009^a^^c^
Polycan:CaLG mixed formula (5:95; mg/kg)				
200	0.893±0.041	0.275±0.026^a^	0.372±0.026^c^	0.115±0.012^a^^c^
100	0.894±0.081	0.269±0.020^a^	0.359±0.030^c^	0.108±0.007^a^^c^
50	0.898±0.051^b^	0.272±0.042^a^	0.351±0.032^d^	0.106±0.017^a^
Polycan:CaLG mixed formula (10:90; mg/kg)				
200	0.907±0.059^b^	0.288±0.018^a^	0.382±0.019^c^	0.122±0.012^a^^c^
100	0.907±0.053^b^	0.278±0.019^a^	0.370±0.018^c^	0.114±0.005^a^^c^
50	0.883±0.054	0.280±0.019^a^	0.357±0.021^c^	0.113±0.009^a^^c^

Values are expressed as the mean ± SD of eight rats.

a P<0.01 and

b P<0.05, vs. sham control;

c P<0.01 and

d P<0.05, vs. OVX control.

BW, body weight; OVX, ovariectomy; CaLG, calcium lactate-gluconate.

**Table II:** Serum osteocalcin and bALP levels after 84 days of repeated oral administration of test materials in osteoporotic rats.

Groups	Serum osteocalcin (ng/ml)	Serum bALP (U/l)
Controls		
Sham	1.24±0.17	1.66±0.21
OVX	2.20±0.27^a^	0.95±0.15^a^
Risedronate sodium	1.58±0.09^a^^b^	1.03±0.15^a^^b^
Single formula (100 mg/kg)		
Polycan	1.88±0.10^a^^b^	1.18±0.18^a^^b^
CaLG	1.77±0.09^a^^b^	1.25±0.09^a^^b^
Polycan:CaLG mixed formula (1:99; mg/kg)		
200	1.72±0.16^a^^b^	1.29±0.14^a^^b^
100	1.79±0.13^a^^b^	1.24±0.12^a^^b^
50	1.88±0.09^a^^b^	1.15±0.13^a^^b^
Polycan:CaLG mixed formula (5:95; mg/kg)		
200	1.66±0.18^a^^b^	1.31±0.15^a^^b^
100	1.74±0.14^a^^b^	1.26±0.11^a^^b^
50	1.84±0.12^a^^b^	1.19±0.11^a^^b^
Polycan:CaLG mixed formula (10:90; mg/kg)		
200	1.55±0.11^a^^b^	1.41±0.12^a^^b^
100	1.63±0.21^a^^b^	1.33±0.12^a^^b^
50	1.75±0.13^a^^b^	1.28±0.15^a^^b^

Values are expressed as the mean ± SD of eight rats.

a P<0.01, vs. sham control;

b P<0.01, vs. OVX control.

bALP, bone-specific alkaline phosphatase; OVX, ovariectomy; CaLG, calcium lactate-gluconate.

**Table III:** Urinalysis after 84 days of repeated oral administration of test materials in osteoporotic rats.

Groups	Dpd (nM)	Dpd/creatinine ratio (nM/g/day)
Controls		
Sham	37.58±3.98	5972.95±746.51
OVX	67.08±3.90^a^	10884.88±1642.09^a^
Risedronate sodium	49.67±5.69^a^^c^	7887.86±966.21^a^^c^
Single formula (100 mg/kg)		
Polycan	54.33±6.00^a^^c^	8839.59±1031.19^a^^c^
CaLG	60.15±6.67^a^^c^	9428.92±1668.14^a^^d^
Polycan:CaLG mixed formula (1:99; mg/kg)		
200	52.68±5.89^a^^c^	8333.03±1034.61^a^^c^
100	56.40±5.66^a^^c^	8833.37±1126.67^a^^c^
50	58.41±5.31^a^^c^	9167.76±1126.57^a^^c^
Polycan:CaLG mixed formula (5:95; mg/kg)		
200	48.92±5.78^a^^c^	7846.84±1210.32^a^^c^
100	54.59±5.50^a^^c^	8824.94±872.25^a^^c^
50	57.48±3.90^a^^c^	9095.29±1056.95^a^^c^
Polycan:CaLG mixed formula (10:90; mg/kg)		
200	47.15±3.12^a^^c^	7492.46±671.09^b^^c^
100	51.04±5.00^a^^c^	8190.17±1198.21^a^^c^
50	53.07±4.72^a^^c^	8567.11±1339.35^a^^c^

Values are expressed as the mean ± SD of eight rats.

a P<0.01 and

b P<0.05, vs. sham control;

c P<0.01 and

d P<0.05, vs. OVX control.

Dpd, deoxypyridinoline; OVX, ovariectomy; CaLG, calcium lactate-gluconate.

**Table IV:** Bone Ca and P contents after 84 days of repeated oral administration of test materials in osteoporotic rats.

Groups	Ca (mg/g bone)	P (mg/g bone)	Ca/P ratio
Controls			
Sham	178.29±8.15	103.08±4.83	1.73±0.02
OVX	120.92±12.96^a^	69.51±7.27^a^	1.74±0.07
Risedronate sodium	148.22±10.61^a^^b^	85.90±7.64^a^^b^	1.73±0.08
Single formula (100 mg/kg)			
Polycan	137.56±6.41^a^^b^	80.12±3.28^a^^b^	1.72±0.02
CaLG	140.73±5.45^a^^b^	79.38±5.68^a^^b^	1.78±0.10
Polycan:CaLG mixed formula (1:99; mg/kg)			
200	148.93±7.73^a^^b^	84.44±4.16^a^^b^	1.76±0.05
100	140.59±8.47^a^^b^	79.82±5.21^a^^b^	1.76±0.08
50	135.22±9.16^a^^b^	78.23±3.97^a^^b^	1.73±0.11
Polycan:CaLG mixed formula (5:95; mg/kg)			
200	153.21±10.77^a^^b^	88.71±7.71^a^^b^	1.73±0.05
100	141.15±7.75^a^^b^	79.75±7.39^a^^b^	1.78±0.11
50	137.39±5.34^a^^b^	78.58±4.49^a^^b^	1.75±0.07
Polycan:CaLG mixed formula (10:90; mg/kg)			
200	161.26±8.53^a^^b^	93.47±3.77^a^^b^	1.73±0.07
100	149.27±8.35^a^^b^	86.21±4.93^a^^b^	1.73±0.03
50	142.49±7.06^a^^b^	81.22±3.21^a^^b^	1.75±0.07

Values are expressed as the mean ± SD of eight rats.

a P<0.01, vs sham control;

b P<0.01, vs. OVX control.

Ca, calcium; P, inorganic phosphorus; OVX, ovariectomy; CaLG, calcium lactate-gluconate.

**Table V:** BMD and FL after 84 days of repeated oral administration of test materials in osteoporotic rats.

Groups	Total	Epiphyseal neck	Mid-shaft	FL (n)
Controls				
Sham	0.1207±0.0026	0.1327±0.0085	0.1017±0.0069	131.11±14.77
OVX	0.1097±0.0050^a^	0.1159±0.0077^a^	0.0914±0.0019^a^	76.27±10.93^a^
Risedronate sodium	0.1326±0.0055^a^^c^	0.1615±0.0188^a^^c^	0.1066±0.0069^c^	104.29±11.51^a^^c^
Single formula (100mg/kg)				
Polycan	0.1169±0.0063^c^	0.1287±0.0029^c^	0.0985±0.0083	97.45±11.55^a^^c^
CaLG	0.1175±0.0034^c^	0.1360±0.0045^c^	0.0999±0.0030^c^	96.89±11.30^a^^c^
Polycan:CaLG mixed formula (1:99; mg/kg)				
200	0.1175±0.0053^c^	0.1433±0.0142^c^	0.1019±0.0046^c^	105.21±12.82^a^^c^
100	0.1172±0.0040^c^	0.1362±0.0059^c^	0.0989±0.0056^c^	96.89±16.03^a^^c^
50	0.1157±0.0057^b^^d^	0.1333±0.0081^c^	0.0983±0.0065^d^	94.48±10.91^a^^c^
Polycan:CaLG mixed formula (5:95; mg/kg)				
200	0.1192±0.0042^c^	0.1437±0.0091^b^^c^	0.1056±0.0095^c^	108.78±10.71^a^^c^
100	0.1181±0.0035^c^	0.1365±0.0080^c^	0.1005±0.0068^c^	94.47±16.98^a^^c^
50	0.1169±0.0054^c^	0.1334±0.0138^d^	0.0982±0.0043^c^	96.12±14.38^a^^c^
Polycan:CaLG mixed formula (10:90; mg/kg)				
200	0.1216±0.0033^c^	0.1418±0.0095^c^	0.1090±0.0109^c^	118.04±11.83^a^^c^
100	0.1201±0.0031^c^	0.1407±0.0079^c^	0.1059±0.0065^c^	109.68±14.94^a^^c^
50	0.1193±0.0063^c^	0.1381±0.0107^c^	0.1020±0.0042^c^	104.87±12.47^a^^c^

Values are expressed as the mean ± SD of eight rats (g/cm^2^).

a P<0.01 and

b P<0.05, vs. sham control;

c P<0.01 and

d P<0.05, vs. OVX control.

BMD, bone mineral density; FL, failure load; OVX, ovariectomy; CaLG, calcium lactate-gluconate.

**Table VI:** Histomorphometry of the femur after 84 days of repeated oral administration of test materials in osteoporotic rats.

Groups	TBV (%)	Tbn (n)	Tbl (mm)	Tbt (μm)	Ocn (n)	OS/BS (%)	Cbt-neck (μm)	Cbt-shaft (μm)
Controls								
Sham	48.36±5.98	28.63±3.78	7.35±0.69	465.03±46.70	8.50±2.45	2.48±0.51	1042.37±137.98	1264.96±215.64
OVX	20.10±2.07^a^	8.25±2.43^a^	3.21±0.40^a^	185.42±28.75^a^	30.63±4.75^a^	22.17±3.81^a^	549.82±82.24^a^	743.76±63.80^a^
Risedronate sodium	53.28±8.94^c^	31.50±3.02^b^^c^	4.83±0.53^b^^c^	174.59±16.86^a^	36.25±7.01^a^	7.11±1.23^a^^c^	782.29±85.97^a^^c^	852.60±80.20^a^^d^
Single formula (100 mg/kg)								
Polycan	36.49±3.77^a^^c^	18.25±1.67^a^^c^	5.19±0.60^a^^c^	271.72±38.72^a^^c^	18.23±2.70^a^^c^	11.55±3.72^a^^c^	788.03±24.83^a^^c^	990.67±107.17^b^^c^
CaLG	32.27±2.46^a^^c^	15.88±1.96^a^^d^	5.37±0.56^a^^d^	305.26±37.91^a^^c^	26.13±3.36^a^	18.05±2.95^a^	860.28±89.76^a^^c^	1037.67±75.58^b^^c^
Polycan:CaLG mixed formula (1:99; mg/kg)								
200	40.80±5.50^b^^c^	20.38±1.69^a^^c^	6.36±0.56^a^^c^	315.14±23.92^a^^c^	14.25±2.92^a^^c^	9.26±1.72^a^^c^	871.51±113.27^b^^c^	1109.18±102.73^c^
100	36.20±4.83^a^^c^	18.38±1.92^a^^c^	5.32±0.41^a^^c^	307.22±16.19^a^^c^	18.50±2.78^a^^c^	13.38±3.90^a^^c^	813.29±65.29^a^^c^	1026.50±76.65^b^^c^
50	29.72±2.74^a^^c^	14.13±1.89^a^^c^	4.91±0.28^a^^c^	289.03±17.61^a^^c^	20.13±3.04^a^^c^	18.46±1.24^a^^d^	753.68±85.50^a^^c^	948.33±67.40^a^^c^
Polycan:CaLG mixed formula (5:95; mg/kg)								
200	46.58±4.50^c^	21.75±1.67^a^^c^	6.47±0.54^a^^c^	322.65±23.11^a^^c^	14.00±4.34^a^^c^	8.48±1.75^a^^c^	947.07±67.10^c^	1129.15±134.17^c^
100	36.82±3.03^a^,^c^	18.63±1.19^a^^c^	5.45±0.68^a^^c^	315.50±23.05^a^^c^	18.25±2.49^a^^c^	12.52±3.98^a^^c^	869.35±82.56^a^^c^	1027.62±78.15^b^^c^
50	33.53±4.80^a^^c^	16.75±1.28^a^^c^	5.23±0.51^a^^c^	293.91±16.64^a^^c^	19.75±2.49^a^^c^	15.70±3.54^a^^c^	772.26±103.37^a^^c^	989.81±77.08^a^^c^
Polycan:CaLG mixed formula (10:90; mg/kg)								
200	50.28±3.06^c^	27.88±2.03^c^	7.70±0.66^c^	395.20±28.97^a^^c^	9.38±1.77^c^	6.78±1.81^a^^c^	995.82±136.21^c^	1186.15±188.42^c^
100	43.46±3.23^c^	23.00±3.38^a^^c^	6.79±0.50^b^^c^	367.72±47.79^a^^c^	13.38±3.74^a^^c^	9.25±1.85^a^^c^	968.71±86785^c^	1126.91±145.68^c^
50	38.10±3.59^a^^c^	18.38±1.77^a^^c^	6.19±0.75^a^^c^	349.57±45.52^a^^c^	17.25±3.85^a^^c^	12.01±2.27^a^^c^	882.55±45.04^a^^c^	1040.69±101.95^b^^c^

Values are expressed as the mean ± SD of eight rats.

a P<0.01 and

b P<0.05, vs. sham control;

c P<0.01 and

d P<0.05, vs. OVX control

TBV, trabecular bone volume; Tbn:, trabecular bone number; Tbl, trabecular bone length; Tbt, trabecular bone thickness; Ocn, osteoclast cell number; OS/BS, osteoclast cell surface/bone surface; Cbt, cortical bone thickness. OVX, ovariectomy; CaLG, calcium lactate-gluconate.
